# Enantioselectivity Effects in Clinical Metabolomics and Lipidomics

**DOI:** 10.3390/molecules26175231

**Published:** 2021-08-28

**Authors:** Regina V. Oliveira, Ana Valéria C. Simionato, Quezia B. Cass

**Affiliations:** 1 SEPARARE-Núcleo de Pesquisa em Cromatografia, Department of Chemistry, Federal University of São Carlos, Rod. Washington Luiz, Km 235, São Carlos 13565-905, SP, Brazil; oliveirarv@ufscar.br; 2 Department of Analytical Chemistry, Institute of Chemistry, University of Campinas, Campinas 13083-970, SP, Brazil; avsimionato@unicamp.br; 3 National Institute of Science and Technology for Bioanalytics, Institute of Chemistry, University of Campinas, Campinas 13083-970, SP, Brazil

**Keywords:** metabolomics, lipidomics, stereoisomers, chiral biomarkers, chiral amino acids, LC-MS, CE-MS

## Abstract

Metabolomics and lipidomics have demonstrated increasing importance in underlying biochemical mechanisms involved in the pathogenesis of diseases to identify novel drug targets and/or biomarkers for establishing therapeutic approaches for human health. Particularly, bioactive metabolites and lipids have biological activity and have been implicated in various biological processes in physiological conditions. Thus, comprehensive metabolites, and lipids profiling are required to obtain further advances in understanding pathophysiological changes that occur in cells and tissues. Chirality is one of the most important phenomena in living organisms and has attracted long-term interest in medical and natural science. Enantioselective separation plays a pivotal role in understanding the distribution and physiological function of a diversity of chiral bioactive molecules. In this context, it has been the goal of method development for targeted and untargeted metabolomics and lipidomic assays. Herein we will highlight the benefits and challenges involved in these stereoselective analyses for clinical samples.

## 1. Introduction

Metabolomics is a rapidly emerging ‘omic’ technology that integrates the study, characterization, and quantification of metabolites in system biology to generate comprehensive profiling for cellular processes [1]. As such, it can accurately and comprehensively provide insights, on a global or network scale, into multiple aspects of the physiological state of a cell or organism and its dynamic responses to genetic variations, environment, and personal lifestyle. Therefore, metabolite profiling offers a readout of an individual’s metabolic phenotype or metabotype, physiological status, and environmental exposure, which can be a major step toward personalized medicine and public health [2]. As the newest omics science, such as genomics, transcriptomics, or proteomics, metabolomics is the systematic study of the metabolome, commonly defined as the complete collection of metabolites, small molecules (with a molecular mass < 1.5 kDa) in living systems, organelle, signaling molecules in biofluids, cells, tissues, and different organisms [3]. Metabolomics has contributed to the discovery of several key disease-related biomarkers, including indicators of pathogenic processes, being considered an extension of clinical chemistry [4]. Hence, metabolomics refers mainly to profiling small-molecule metabolites, while biomarkers are objectively measured as indicators of biological or pathogenic processes [3], which can include small molecular entities, as well as large molecular weight proteins and genetic materials.

Lipidomics has been described as a branch of metabolomics especially devoted to lipids analysis for their characterization and attribution of molecular functions [5]. Lipids are simply defined as hydrophobic biological molecules such as fatty acyls, eicosanoids, glycerophospholipids, sphingolipids, and others [6].

Metabolomics and lipidomics methodologies fall into two recognizable approaches: nontargeted and targeted analysis [7]. Nontargeted metabolomics/lipidomics is the comprehensive analysis of all measurable unknown metabolites/lipids in a given sample. This approach provides semi-quantitative data, meaning that peak areas are reported for each metabolite/lipid instead of absolute concentrations, which allows the assessment of the relative abundance of the detected metabolites/lipids between experimental groups in different conditions or across a population. As opposed to the nontargeted approach, the targeted metabolomics/lipidomics include the quantitative determination of chemically characterized and biochemically annotated metabolites by using authentic chemical analytical standards and calibration curves [7].

The metabolome and lipidome are very complex, being composed of a variety of chemically diverse achiral and chiral molecules, which are called primary metabolites and are routinely produced by endogenous catabolism or anabolism. The metabolome can be divided into the primary metabolome, which is controlled by the host genome, and the co-metabolome that is dependent on the microbiome [8]. Recently, several studies have also shown the connection between microbiome and lipidome and how microbial lipids alter intestinal and circulating lipid concentrations in the host cholesterol and sphingolipid homeostasis, thus impacting human health [9]. The integration of metabolomic and lipidomic analysis provides a comprehensive overview of the metabolic network enabling the identification of critical metabolic drivers in disease pathology, facilitating the study of the interaction between lipids and metabolites as the disease progresses [10]. In this regard, enantioselective analysis has been recognized as of great significance for disclosing chiral biomarkers and their diagnostic and prognostic clinical value. Herein we will outline current liquid chromatography (LC) and capillary electrophoresis (CE) protocols that have been pursued either for targeted or nontargeted metabolomics and lipidomics chiral analysis with their pros and cons for clinical samples.

## 2. Chiral Metabolites and Lipids Separation by Liquid Chromatography

### 2.1. Amino Acids

It is by now well established that d-amino acids are biomarkers of diseases with diagnostic value [11,12,13,14]. The distribution, function, and physiological role of these amino acids have been the motif of several research projects in the last decade.

Despite this, stereoisomers are still overlooked in various tissues and/or physiological fluids, in peptide characterization experiments, and in the metabolomics process, thus demanding stereochemical analytical protocols [15,16,17].

The analysis by LC of the enantiomeric ratio (e.r.) of amino acids (AAs) still relies frequently on the indirect approach by derivatization using reagents such as 1-fluoro-2,4-dinitrophenyl-l-alanine amide (Marfey’s reagent, FDAA), 1-(9-fluorenyl)ethyl chloroformate, *ortho*-phthaldialdehyde/*N*-acetyl-l-cysteine, and (*R*)-1-Boc-2-piperidinecarbonyl chloride, with separation of the formed diastereomeric mixture by achiral columns, mostly under reverse elution mode [18,19]. The weakness of the indirect chiral analysis is primarily due to reaction kinetics and impurities of derivatization reagents that can lead to inaccuracies. Problems related to racemization due to pH or temperature also require special attention [20,21].

**Scheme 1:**
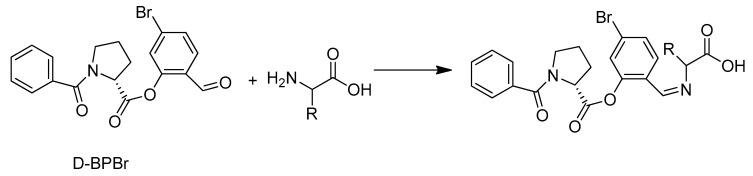
D-BPBr derivatization reaction with amino compounds [22].

In this regard, a remarkable advance has been recently published, with the synthesis of a chiral bromine isotope probe: 1-benzoyl-pyrrolidine-2-carboxylic acid 5-bromo-2-formylphenyl (2*R*)-1-benzoylpyrrolidine-2-carboxylate (d-BPBr) [22]. This probe has shown stability, reactivity, and chiral selectivity towards D-AAs. The stereoselectivity was credited to the nonrotational chiral center of the proline moiety and the salicylaldehyde ring. The reaction of the probe D-BPBr with an enantiomeric mixture of AAs forms Schiff’s bases (Scheme 1) that can be resolved as a diastereoisomeric pair on C18 columns and can be quantified either in the positive or negative mode with a pair of ions having similar abundance and 2-Da mass difference. The developed method has been shown to be useful for targeted and nontargeted analysis and might be handy to detect D-AAs as biomarkers with diagnosis values.

For direct liquid chromatography (LC) separation of amino acids in complex biological mixtures, using chiral stationary phases (CSPs), it is important to pay attention that only amino acids containing a chromophore group, such as phenylalanine, tyrosine, and tryptophan can be detected by an ultraviolet/visible light detector (UV-Vis). Because of this, it is common to see derivatization of the most reactive functional moiety of the AAs to introduce a chromophore or a fluorophore group, increasing the detection sensitivity [14]. The derivatization can also be used to increase resolution between acidic, basic, and polar amino acids. The use of detectors such as evaporative light scattering detector (ELSD) and Corona Charged Aerosol Detector (CAD) have been reported, but it is expected that mass spectrometry (MS) will be the detector of choice [23,24].

An interesting application is the simultaneous quantification of 19 enantiomeric pairs of proteinogenic AAs and the achiral glycine in serum, from hepatocellular carcinoma patients and healthy individuals, which was achieved with a 13 min run using a CROWNPAKCR-I(+) chiral column. The study disclosed d-glutamate and d-glutamine as the most downregulated serum markers. Owing to the same selected reaction monitoring transitions (SRM) and chromatographic co-elution, l-glutamine and d-lysine were not differentiated, and d,l-proline were not resolved [25].

The chiral analysis of AAs is impaired by the structural isomers and diastereoisomers present in the mixture, and their separation must be achieved in the chromatographic space since the co-elution cannot be perceived by MS. Another drawback for measuring e.r. is the higher proportion of the l-enantiomer [21,25].

The lack of a chiral isotopically labeled internal standard has been another serious problem in tackling the matrix effect in quantitative chiral analysis by liquid chromatography coupled to mass spectrometry (LC-MS). To overcome this issue, a protocol to produce a tailored made internal standard with the desired d-amino acids level has been described together with a chromatographic procedure for AAs chiral separation with a zwitterionic Chiralpak ZWIX(+) column. These protocols should impact metabolomics analysis [26].

A weak anion-exchange-type chiral column (Chiralpak QN-AX column) was connected in series to a zwitterion-exchange type chiral column (Chiralpak ZWIX(+) or ZWIX(−) column) and used for the resolution of a series of AAs which were detected in tandem MS/MS in a 20 min run. The diversity of molecular interactions of the two CSPs in series allowed the chemo- and enantioseparation of the target AAs. With this assay, samples from a cohort of 305 women classified as control, mild-cognitive-impairment, and dementia groups were examined, and d-proline was correlated with early cognitive decline [27].

Due to the higher chromatographic selectivity, online 2D LC has been thought for measuring e.r. of target AAs. In these configurations, the AAs are usually separated at an achiral column and then transferred to the second dimension for chiral resolution [21]. While for a single heart-cutting approach (LC-LC) the chromatography space is not relevant, for multiple heart-cutting (mLC-LC), selective comprehensive 2D-LC (sLC × LC), and comprehensive 2D-LC (LC × LC) the high peak capacity is an important goal [28]. The advances in new valve technologies and ultra-fast LC chiral columns [29,30] produced the means for two-dimensional liquid chromatography (2D LC) in metabolomic studies for enantioselective AAs measurement [13].

Although heart-cutting LC-LC using a chiral column in the second dimension is well established [31], only recently, applications of mLC-LC and comprehensive LC × LC have been growing, due to mainly two factors: commercial 2D LC systems and ultra-fast LC chiral columns. The latter is of paramount importance in designing the experiments [32]. The second-dimension column needs to elute very fast to permit multiple transfers in a minimum time. Coreshell technology and small particles size can furnish this support, but the choice of the chiral selector is still the Achilles heel. In the case of large retention differences, in the second dimension, which usually is the case of complex AAs mixtures, the design of the elution conditions in a single run can impact the resolution [23].

An innovative approach has been described for providing direct stereochemical information of peptides samples [33]. To meet this end, a chiral × chiral 2D LC system was designed using two CSP of the same type but of opposite stereochemistry (quinine and quinidine carbamates). The orthogonality presented merely by the stereocenter of the CSP resulted in the inversion of elution order in the two dimensions. The use of ultra-fast Coreshell columns allowed LC × LC fast run. In the contour plot, the achiral components and impurities were lined up on the diagonal line in the 2D separation space facilitating the stereochemical profiling of the AAs. The AAs detection was carried out by fluorescence as *N*-fluorenylmethoxycarbonylated amino acids derivatives. The developed method has been used for the analysis of hydrolyzed bacitracin sample, which is a cyclic peptide produced by *Bacillus subtilis* via nonribosomal peptide synthetases and has clearly differentiated the d-amino acids in the sample. The authors point out that the protocol can be applied for unknown complex samples but the lack of diverse commercial CSP with opposite stereochemistry may limit the general use [33].

For achieving higher resolution, a 3D LC system has been designed for measuring trace levels of d-Asn (asparagine), d-Ser (serine), d-Ala (alanine), and d-Pro (proline), possible biomarkers of chronic kidney disease (CKD) in the human plasma. For fluorescence detection, the AAs were pre-column derivatized with 4-fluoro-7-nitro-2,1,3-benzoxadiazole (NBD-F). The target AAs were separated in the first and second dimensions from interfering matrix compounds by the differences in hydrophobicity and anionic strength, using a reversed-phase and anion-exchange column, respectively. The chiral AAs resolution was attained at the third dimension with a Pirkle type KSAACSP-001S column. The three-dimension run was operated in a simultaneous format, which produced a total analysis time of 180 min. The validated method was applied to measure the targets AAs in plasma samples of patients of CKD and of healthy volunteers. The target D-AAs were found in all patients’ samples and the percentage of D-values for Asn and Ser showed a good correlation with the estimated glomerular filtration ratio of the patients [34].

### 2.2. Hydroxycarboxylic Acids

Chiral hydroxycarboxylic acids (HAs) have been related as biomarkers to some, but not only, metabolic diseases [35,36,37]. As with the amino acids, the ability to quantify the HAs enantiomers in the metabolome is essential [38,39]. For instance, d- and l-2-hydroxyglutaric acid (d-2-HG and l-2-HG) are normal endogenous metabolites, but they are biomarkers of the three inborn acidurias (d-2HGA, l-2HGA, and d,l-2HGA), a disease that in the prime of life causes serious disability [35]. These 2-HGs are also oncometabolites and their activities are beyond epigenetic control and might be involved in the normal process of T cell regulation [40].

Methods for enantioselective separation of 2-hydroxy and 3-hydroxy-derivatives have been described using chiral columns either by gas chromatography coupled to mass spectrometry (GC-MS) [41] or liquid chromatography coupled to mass spectrometry (LC-MS) [36,42,43] approaches. The separation of 3-HAs, from hexanoic to myristic acid, were separated in a CHIRALPAK IA-U (amylose *tris*(3,5-dimethylphenylcarbamate) immobilized onto a 1.6 mm silica) with resolution increasing with the chain length. The gradient elution allowed the separation of the series in a 20 min run. The developed method was used for the analysis of 3-HAs released from a novel class of lipopeptides. Rhamnolipid (R-95) (bacterial surfactant) hydrolysate was employed as a standard for disclosing the absolute configuration of the 3-HAs side chain of a new lipopeptide [43].

**Scheme 2:**
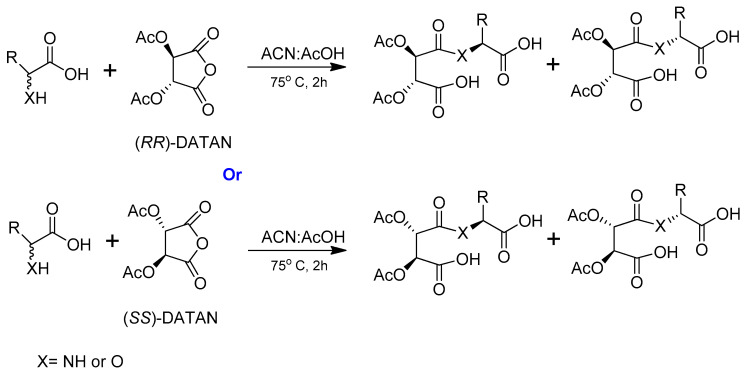
Derivatization reaction of HAs and AAs with either (*RR*)-DATAN or with (*SS*)-DATAN to form their respective diastereomeric mixtures [39].

For nontargeted indirect chiral analysis, an LC-HRMS method has been described based on the simultaneous derivatization of −OH/−NH_2_ moiety-containing metabolites, including HAs and AAs, with the enantiomeric pair of diacetyl-tartaric anhydride (DATAN) [39]. The diastereomers were identified by a diagnostic reagent fragment ion with a data-independent acquisition (DIA) mode. To differentiate achiral metabolites from the chiral ones, aliquots were prepared with either (*RR*)-DATAN or (*SS*)-DATAN (Scheme 2). The achiral metabolite derivatives did not change their retention time in respect to the used DATAN enantiomer, while the chiral metabolites formed a diastereomeric pair eluting at different retention times. Reversal of the diastereomer elution time, based on the used DATAN enantiomer, served as a diagnostic tool for identifying the enantiomers of HAs and AAs. The developed method produced the separation of 214 chiral compounds including 106 AAs and 28 HA from a 301-metabolite standard library, in a single analytical run [39].

Bone marrow and peripheral blood plasma samples from patients with acute myeloid leukemia were screened to observe the influence of the induction phase of chemotherapeutic treatment on the chiral metabolites by comparing with samples collected at diagnosis. The method was able to detect trace levels of d-enantiomers of HAs and AAs, and some of them were significantly altered [39].

### 2.3. Lipids

Lipids perform many different functions in a cell and are fundamental building blocks of life, also have a remarkable structural and functional diversity [6], and are present in a broad range of concentration levels, which makes their comprehensive analysis very challenging. The selectivity, specificity, sensitivity, and speed of mass spectrometry (MS) in combination with its recent technology advances have contributed to the development of mass spectrometry-based lipidomics. Different targeted and nontargeted LC-MS strategies have been optimized and developed for comprehensive lipid analysis, including shotgun lipidomics [44]. In essence, the vast majority of analytical methods focus on achiral determinations being unable to distinguish between enantiomers and, as previously highlighted for amino acids, lipids stereoisomers evaluation is still in less numbers and of high importance [45].

Methods for the enantioselective separation of oxylipins for the analysis of autoxidized fatty acids and platelet releasates were described using a CHIRALPAK IA-U and CHIRALPAK IC-U polysaccharide chiral columns with sub-2 μm particles and immobilized amylose *tris*(3,5-dimethyl-phenylcarbamate) as the chiral selector [46]. Oxylipins are metabolic intermediate from oxygenated polyunsaturated fatty acids (PUFAs) and are biomarkers of inflammatory processes. They are chiral HAs with the hydroxy position far away from the carboxylic moiety and different regio-chemistry. The enantioselective analysis of oxylipins allows the differentiation in their formation regarding enzymatic (cyclooxygenase -COX, lipoxygenase -LOX, and cytochromeP450) or oxidative stress origin [46,47]. Polysaccharide-based chiral phases have been demonstrated to be useful for LC-MS/MS comprehensive profiling regio- and stereoisomeric oxylipins in biological and clinical samples, under reverse elution mode [46,47].

A different approach employed for chiral LC-MS of oxylipins analysis is a normal-phase with a nonpolar mobile phase (e.g., a mixture of hexane and organic modifier); however, a disadvantage of this mobile phase composition is its negative impact on the electrospray ionization (ESI) process, resulting in poor sensitivity. To circumvent this problem, derivatization of bioactive lipids with pentafluorobenzyl bromide (PFB-Br), an electron-capturing group, can be used followed by atmospheric pressure chemical ionization (APCI). This technique is called electron capture (EC) APCI-MS was used by Mazaluskaya et al. for the target chiral lipidomic analysis of hydroperoxyeicosatetraenoic acids (HETEs) in human serum and plasma [48]. The method employed a CHIRALPAK AD-H column and the mobile phase was a mixture of hexane (A) and 2-propanol/methanol (1:1, *v*/*v*) (B) making use of a gradient elution mode. High LC-MS sensitivity was obtained by monitoring the fragment ions of the pentafluorobenzyl derivatives of the oxidized lipids during the dissociative electron capture process in negative ionization mode, followed by ion-trap analysis promoted by the Q-Exactive HF Hybrid Quadrupole-Orbitrap mass spectrometer [48]. Besides the use of PFB to increase the sensitivity of lipids when using APCI as the ionization source and normal elution mode chiral chromatography, the use of targeted stable isotope dilution liquid chromatography-mass spectrometry (SID-LC-ECAPCI-MS) allows for accurate and precise quantification of stereoisomers of oxidized bioactive lipids, since this procedure will account for sample loss during sample preparation as well as matrix effects during ionization process [49].

An excellent example of practical application is the pharmacological characterization of three commercially available platelet 12-lipoxygenase (p-12-LOX) inhibitors on the generation of prostanoids and HETEs in human whole blood and plaletes-rich plasma, which was properly conducted using targeted chiral lipidomic analysis and a triple quadrupole as a mass analyzer [50]. The chiral study aimed to characterize the biosynthesis of eicosanoids (thromboxane-TXB2, prostaglandins-PGE2 and HETEs) induced in clotting whole blood by endogenously generated thrombin. The enantioselective separation of 15*R*- and 15*S*-HETE allowed assessing the enzymatic product of COX activity, while the levels of 12*R*-HETE, 8*R*-HETE, 8*S*-HETE, and 5*R*-HETE were used to evaluate the nonenzymatic oxidation of the acetylsalicylic acid. Then, 5*S*-HETE concentrations were determined as a product of the 5-LOX specific activity.

As previously discussed, the separation of 3-hydroxy fatty acids was conducted by direct chiral LC-MS without derivatization using a CHIRALPAK IA-U column [43]. Differently from other lipid methods, the chromatographic condition was a reversed-phase and gradient elution condition with both acetonitrile and methanol as organic modifiers, covering hydrocarbon chain lengths between C6 and C14. Elution orders were derived from rhamnolipid (*R*-95) as enantiomer standard or enriched (*R*)-3-hydroxy fatty acids recovered after ester hydrolysis. The *S*-configured acids were consistently eluted before the respective *R*-enantiomers [43].

A multi-targeted chiral lipidomic method for glycosphingolipids (GSLs) has also been described. GSLs consist of a hydrophilic carbohydrate structure attached to a lipid tail that contains the hydrophobic ceramide, found in the outer leaflet of the plasma bilayer membrane, and is associated with the pathogenesis of diverse diseases. Due to the amphiphilic nature of the GSLs, their chromatographic separation in either the normal or reversed phase is very challenging, and the species cannot be efficiently separated. Therefore, Fujiwara et al. used the conformation structures of GSLs and evaluated their separation by chiral chromatography [51]. An innovative aspect of the developed method was its ability to simultaneously quantify different classes of GSL such as ganglioside, sulfatides, and neutral GSLs, which was accomplished by carrying out collision-induced dissociation (CID) experiments of individual GLS types in the positive and negative ion modes by varying the collision energy (CE). The authors investigated six chiral columns including CHIRALPAK IA-3, IB-3, IC-3, ID-3, IE-3, and IF-3 under reversed-phase elution-mode. The results indicated that the chromatographic resolution of the GSL species based on the separation of the ceramide and sugar moieties, in association with the best MS sensitivity, was obtained with the CHIRALPAK IF-3 chiral column.

Effect of alcohol modifier as part of mobile phase composition for the enantiomeric separation of hydroxyeicosanoids by chiral columns was also investigated [52]. It is well known that cellulose and amylose derivatives are the most widely used chiral selectors for enantiomeric separations and they provide improved enantioselectivity when using normal phase mode composed of hexane and alcohol as the organic modifier. When the CHIRALPAK AD column, a *tris*-(3,5-dimethylphenyl carbamate) derivatized amylose support, was evaluated for a variety of lipoxygenase products and other hydroxy derivatives using mixtures of hexane, and changing the alcohol modifier from isopropanol to ethanol or to methanol, great improvements in the enantiomeric separations as well as in the chromatographic efficiency were observed, with higher theoretical plates and sharper chromatographic peaks, increasing the sensitivity [52]. The only limitation is that the proportion of methanol to hexane should be kept at 5% to avoid a biphasic mixture. On the other hand, when the same approach was tested with the CHIRALPAK OD column, a *tris*-(3,5-dimethylphenyl carbamate) derivatized cellulose support, no improvement was noticed.

Although the majority of chiral separations of oxylipins have been performed on polysaccharide-based chiral stationary phases, to some extent Pirkle-type chiral stationary phases have been also an option for this class of lipids. Enantiomers of hydroperoxyl fatty acids HOMEs, HpOME(E)s, and HpOME(Z)s were separated with Pirkle-type chiral stationary phase Reprosil chiral NR^2^ and NR using LC-MS^3^ strategies [53].

Chiral lipidomics analysis is in its great majority conducted for targeted determinations considering a selected class of bioactive lipids from polyunsaturated fatty acids, such as derivatives of arachidonic acids (AA), linoleic acid (LA), eicosanoic acids, and stearidonic acid [54,55]. The polysaccharide-based CPSs are the first choice for these enantiomeric separations, with regard to *tris*-(benzoates), *tris*(phenylcarbamates) of cellulose and amylose, and *tris*(5-chloro-2-methylphenylcarbamate)-amylose-based selectors. A recent review describes in detail the enantioselective analysis of oxygenated polyunsaturated fatty acids using the polysaccharide-based chiral stationary phases [56].

The field of chiral lipidomics is in a continuous search and improvement to further provide more and better enantioresolution and enantioselectivity for complex mixtures of stereoisomers. In some cases, derivatization with pentafluorobenzyl bromide (PFB-Br) is adopted to provide better sensitivity when using normal elution and APCI ionization. During lipidomic analysis, other analytical strategies are also explored to improve selectivity and sensitivity during the data acquisition, which includes selected reaction monitoring (SRM) and multiple reaction monitoring (MRM) experiments for targeted lipidomics, and product ion scanning, precursor ion scanning, and neutral ion scanning for focused lipidomics [57].

## 3. Chiral Metabolites Separation by Capillary Electrophoresis

Capillary electrophoresis (CE) has been widely used for the separation of chiral compounds due to the technique’s versatility, high efficiency, and low sample/background electrolyte (BGE) consumption. Therefore, it is considered a green technique, which furnishes complementary information in metabolomic and lipidomic multiplataforms investigation, since it is mainly focused on the separation of ionic or partially ionizable molecules [58,59]. Since the first report by Richard Zare and co-authors in 1985, a landmark for chiral CE, chiral selectors’ additives, and other analysis conditions have been continuously improved [60,61,62,63]. The 1985 pioneer work demonstrated separation of d,l-amino acids by CE with laser induced fluorescence detection (CE-LIF). For this purpose, analytes derivatization with a dansyl group has been performed to induce fluorescence. The novelty of this work resided on the composition of the BGE (l-histidine and Cu(II) at pH 7.0—a chiral support electrolyte), so that diastereomeric complexes were differently formed with d,l-dansyl amino acids, resulting in a fast separation (less than 10 min) with lower detection limits than chiral LC methods [64]. From then on different chiral BGE additives have been used for enantiomers separation by CE, such as cyclodextrins (CD) [65,66], modified crown-ethers [67], antibiotics [68], chiral surfactants [69,70], proteins [71], ionic liquids [72,73], linear and cyclic saccharides [74], chiral nanoparticles [75], and others [76].

Unlike the most used separation mode on capillary electrophoresis—capillary zone electrophoresis (CZE), where analytes are separated according to charge-to-size ratio—chiral CE is based on the differences of enantiomers mobilities after interaction with a chiral selector, since the original enantiomeric pair presents essentially the same charge and mass. Therefore, in an achiral sample/background electrolyte (BGE), no separation is observed. Furthermore, when using another CE separation mode widely applied as well—micellar electrokinetic chromatography (MEKC), where analytes are separated according to the interaction with the micelle hydrophobic inner and the aqueous BGE or the interaction with the micelle charged core and the aqueous BGE (constituting a dynamic equilibria)—enantioseparation also requires a BGE containing a chiral additive, since the hydrophobic and charge properties of the enantiomeric pair are approximately the same.

The addition of chiral selectors to the BGE in chiral CE constitutes one of the main advantages of this technique, since small amounts of this compound are required to perform high-resolution separation, allowing the evaluation of different chiral selectors. This is especially important when testing expensive chiral selectors. Besides, mixing the chiral selector with the BGE, makes chiral CE a versatile technique.

Nevertheless, the poor detectability of CE constitutes one of the main drawbacks of this technique, due to the low amount of introduced sample (in the order of nanoliters—nL) and the small optical path length when optical detection is used. This limitation is even more pronounced when chiral separations of metabolites present in biological samples and/or metabolomics analyses are performed, although metabolites are found in a vast concentration range in biological samples (from pmol/L to mmol/L) [77]. To overcome this limitation, some strategies have been employed, such as optimization of sample preparation and analytes pre-concentration, as well as the use of capillary-end detection systems such as laser-induced fluorescence detection (LIF) and MS [63,78].

Enhancing metabolites detectability by sample pre-concentration has been often used in chiral CE. Additionally, on-line pre-concentration also provides peaks narrowing, enhancing separation efficiency. On-line platforms are adequate strategies, since they may provide sensitivity, enantioselectivity, high analytical frequency, and fast analyses.

**Figure 1:**
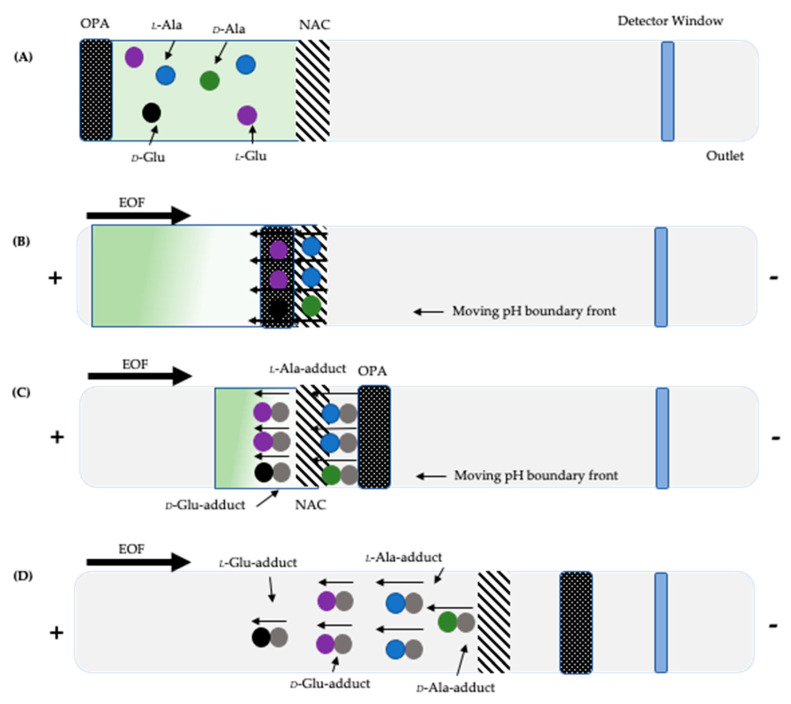
General scheme of SPDE-CE for chiral amino acids separation. (**A**) multiple hydrodynamic injection sequence; (**B**) on-line sample pre-concentration by dynamic pH junction and transient isotachophoresis; (**C**) in-capillary chemical labeling by zone passing of OPA/NAC; (**D**) chiral separation of diastereomeric amino acid adducts enantioselectivity by SPCD–CE. Adapted from [81].

On-line sample pre-concentration and chemical derivatization (SPCD) for chiral CE has been performed in a single capillary. Britz-McKibbin et al. state that, in this case, the capillary has simultaneous functions of pre-concentrator, micro-reactor, and chiral selector [79]. Firstly, introduced for the analysis of phosphoamino acids, such as P-Ser, a long length sample plug (dissolved in a BGE with pH 6.5) was introduced into the electrophoretic capillary already filled with borate BGE pH 9.6 to promote sample stacking by dynamic pH junction. Such a strategy is successful for weak acid and weak bases metabolites since analytes mobilities are sufficiently altered within the BGEs acidities, i.e., the running and the sample BGEs. The same capillary system was used for on-line analyte labeling with 9-fluorenylmethyloxycarbonyl chloride (FMOC), to promote UV detection. For this purpose, FMOC was also introduced into the capillary, after sample plug and a short spacer plug of running BGE, in order to avoid zone mixing and provide adequate pH for reaction. Upon electric field application, the sample plug was narrowed, causing analyte concentration and a subsequent reaction with FMOC [80]. Afterward, the same SPCD-CE principle was applied to the analysis of d-amino acids in *Escherichia coli* culture medium to evaluate extracellular amino acids flux (uptake/release). FMOC was replaced by *ortho*-phthalaldehyde/*N*-acetyl l-cysteine (OPA/NAC) due to rapid amino acid enantiomers reaction in a basic BGE to form diastereoisomeric amino acids (isoindole adducts) with suitable UV absorbance (Figure 1). Under this configuration, the addition of 1 mmol/L of β-CD to the running BGE formed inclusion complexes with the amino acids adducts, allowing separation of d,l-Asp, d,l-Ala, d,l-Glu, but not d,l-Ser, which co-migrated with d,l-Asp. However, as the goal of this work was the separation of peptidoglycan from the *E. coli* cell wall (containing mostly d-Ala and d-Glu) a simpler BGE (without the addition of β-CD) was successfully used [81]. Kühnreich and Holzgrabe have also used OPA/NAC for the in capillary amino acids derivatization, successfully separating 16 chiral amino acids by CE-UV, namely: alanine, arginine, asparagine, aspartic acid, glutamine, glutamic acid, histidine, isoleucine, leucine, lysine, methionine, phenylalanine, serine, threonine, tryptophan, tyrosine, and valine. β or γ-CDs were added to the BGE to achieve separation. The authors stated that proline and cysteine derivatization was not possible due to the secondary amine group of the former and the thiol moiety of the latter [82].

The potentiality of SPCD-CE has been thoroughly demonstrated for amino acids analyses since d-amino acids have already shown important physiological roles [32]. More specifically, d-Asp has been described as an important mammals’ neuromodulator or neurotransmitter [83] and has been already reported in elevated levels in the cerebrospinal fluid of Alzheimer’s patients [84], while diminished in brain tissues, contributing to a lower *N*-methyl-d-aspartate (NMDA) receptor function, which implies to memory deficits [85]. On the other hand, d-serine was found at a 25% decreased level in the CSF of schizophrenia patients compared to healthy individuals [82,86].

Accumulation of short-chain hydroxy acids in body fluids is related to some human diseases. Lactic acid isomers are the product of carbohydrate metabolism. However, while l-lactic acid is formed from pyruvic acid glycolysis, d-lactic acid is found in minute concentrations, but may be produced from the intestine flora under pathological conditions or from methylglyoxal metabolism. Nevertheless, mammals do not absorb d-lactic acid as well as l-lactic acid, and a high concentration may be associated with type 2 diabetes mellitus [87], short bowel syndrome [88], acute intestinal ischemia [89], schizophrenia [90], kidney injury [91], appendicitis [92], and other diseases [88].

CE-UV with direct detection at 200 nm has been successfully applied to the determination of d,l-lactic acid in amniotic fluids, plasma, urine, and cerebrospinal fluid. For this purpose, three relevant factors (evaluated by a Pareto Chart) were optimized by a multivariate method, namely: chiral selector concentration (2-hydroxypropyl-β-cyclodextrin) BGE pH, and BGE concentration (phosphate buffer). Since analytes are mostly anionic under the optimum BGE pH (6.0), electrode polarities were inverted (sample introduction at the cathode end, and detection occurring close to the anode end), allowing analytes detection. Although this method was applied to biological fluids analyses, further investigation with a larger sample cohort, including diseased individuals, must be performed to evaluate the method potential on chiral metabolomics analyses [93].

l-Pipecolic acid is an important biomarker for peroxisomal disorders [94], while d- and l-enantiomers are found at discreetly higher concentrations in individuals with liver cirrhosis or chronic hepatic encephalopathy [95]. Separation of d,l-pipecolic acid has been performed by MECK with diode array detection upon previous off-line derivatization with FMOC, in order to introduce a chromophore group into the analytes structures. The addition of 1% (*w/v*) of a polymeric surfactant (poly(sodium *N*-undecanoyl-l,l--leucyl-valinate)-poly-l,l--SULV) to the BGE provided suitable enantiomeric separation within 16 min. However, the authors stated that such compounds were not commercially available and, since a high amount was required to achieve the expected separation, other chiral selectors were investigated. The BGE composition was optimized and resulted in the pioneering use of an ionic liquid (d-alanine tert-butyl ester lactate-d-alaC4NTf2) to improve enantiomeric resolution in CE. More specifically, separation occurred with a resolution of 1.87 using a BGE composed of 40 mmol/L borate (pH 9.5), 30 mmol/L sodium dodecyl sulfate (SDS), 30 mmol/L β-CD, 15% isopropanol, and 10 mmol/L d-alaC4NTf2. The method was not applied to real samples, but biological fluids of diseased patients submitted to appropriate preparation and pre-concentration could be successfully analyzed by this method in order to diagnose the previously pointed metabolic disorders [96].

To address some CE issues, such as the low detectability (especially when optical detectors are used) and selectivity, the hyphenation with MS has been increasingly used [97]. However, the flexibility of BGE composition is constrained in CE-MS methods, since the CE effluent has to be carefully considered to maintain MS integrity. Therefore, BGE composition must be restrained to volatile or semi-volatile species, particularly when a sheathless interface is used. Towards chiral CE-MS analysis, the application to endometabolites in human biofluids samples is still scarce. Somsen et al. have derivatized d,l-amino acids with FMOC to improve electrospray (ESI) efficiency and augment analyte mass, in order to avoid the mass spectrometer noise from the low mass range. Besides, amino acids’ reaction with FMOC takes only some minutes, which is a significant advantage towards analytical frequency when compared with other derivatizing agents. For a suitable separation/detection, BGE and sheath liquid compositions, as well as ESI parameters were thoroughly optimized, resulting in a BGE composed of 50 mmol/L ammonium bicarbonate at pH 8, containing 15% isopropanol (*v/v*) and 10 mmol/L β-CD, while the sheath liquid was comprised by isopropanol:water:1 mol/L ammonium bicarbonate (50:50:1, *v/v/v*). Within the 20 d,l-amino acids analyzed, 15 showed enantioseparation resolution higher than 0.5, and 9 presented resolution better than 1.2, although d,l-arginine, d,l-alanine, d,l-tyrosine, and d,l -lysine could not be separated. The optimized method was applied to the analysis of amino acids enantiomers in CSF since it has been already reported as indicative of some neurological disorders. Before the derivatization of amino acids with FMOC, the CSF had to be prepared by a simple step of protein precipitation with cold acetonitrile. The method showed adequate separation of 12 d,l-amino acids, although with LODs in the low µmol/L range [98].

An interesting alternative of d,l-amino acids analyses by CE-MS has been proposed by Prion et al. using 1-(9-fluorenyl) ethyl chloroformate (FLEC) as a labeling agent. To improve method selectivity, MEKC mode has been selected using ammonium perfluorooctanoate (APFO) as a semi-volatile surfactant. 14 chiral amino acids were fully separated with limits of detection (LODs) at the nmol/L range. Blank CSF samples were analyzed showing the separation of d,l-serine and d,l-glutamine with an enantiomeric ratio around 4.8–8.0% and 0.34–0.74%, for d-serine and d-glutamine, respectively. According to the authors, the levels of such d-amino acids in Alzheimer’s patients may be higher than the method detectability, showing a putative application [99]. The same research group has also used the same method with d,l-amino acids in-line derivatization. Although initial applications to d,l-amino acids spiked CSF analyses were performed by the optimized method, only 8 d,l-amino acids were fully separated (Resolution > 1.5), while 6 d,l-amino acids were partially separated (R > 1.0), and method detectability was in the µmolar range. Since the mass analyzer was an ion trap (IT), such low detectability would probably be improved using a more sensitive equipment, like a triple quadrupole (QqQ) or a quadrupole-time of flight (QToF) [100].

Hence, there is room for CE-MS metabolites chiral separation improvement, since pioneer works have already shown method potentiality. Such improvements may reside in the use of sheathless interfaces (to prevent CE effluent dilution with consequent detectability enhancement), analytes on-line pre-concentration, and the investigation of novel BGE additives compatible with the MS hyphenation.

## 4. Conclusions

Metabolomics and lipidomics are two emerging technologies offering good prospects for a comprehensive biological systems-level study of a wide range of metabolites and lipids. Improvements in chiral analysis for complex mixtures of metabolites and lipids in association with advances in MS technologies have greatly enhanced the developments and applications of these two ‘omics’ approaches.

The present work also demonstrated that chiral separation by CE seems to be an interesting analytical approach for screening analysis and quantification of bioactive molecules present in different matrices.

In conclusion, chiral metabolomics and lipidomics analysis are powerful tools to uncover the enantiomeric signature of the end products of various biochemical processes catalyzed by enzymes, allowing an useful molecular insight into an organism’s biochemistry, leading to the discovery of new and specific biomarkers and/or to a better understanding of a disease process. The integration of chiral separation and MS detection are requirements in the enantioselective field to improve further achievements regarding deciphering mechanisms of metabolites and lipids-mediated diseases.
